# Loneliness and problematic pornography Use: What is the role of emotion regulation and interaction with content Creators?

**DOI:** 10.1016/j.abrep.2024.100550

**Published:** 2024-05-03

**Authors:** Maria Vescan, Mal Flack, Kim M Caudwell

**Affiliations:** a Faculty of Health, Charles Darwin University, Ellengowan Drive, Brinkin NT 0810, Australia; b Researchers in Behavioural Addictions, Alcohol, and Drugs (BAAD), Charles Darwin University, Ellengowan Drive, Brinkin NT 0810, Australia

**Keywords:** Pornography, Loneliness, Emotion regulation, Problematic pornography use, Content creator, Content creation platform

## Abstract

Problematic pornography use is a growing area of interest necessitating a deeper understanding of its contributing factors. Loneliness and emotion regulation difficulties have been advanced as factors that may exacerbate or perpetuate PPU, however, to date, researchers typically consider these factors in isolation. In addition, the evolving landscape of problematic pornography is further shaped by the emergence of pornography content creators. This study explores the relationships between loneliness, emotion regulation difficulties and problematic pornography use and individuals’ interactions with pornography content creators. Specifically, we examine whether emotion regulation difficulties mediate the relationship between loneliness and PPU and whether this mediation is contingent on interactions with content creators. A total of 213 adults (*M*_age_ = 35.57 years, *SD* = 16.90; 77.50 % men) participants completed an online questionnaire assessing PPU, emotion regulation difficulties and loneliness. Results revealed loneliness is associated with increased PPU and emotion regulation difficulties. Emotion regulation difficulties, in turn, relate to PPU. Though interaction with pornographic content creators was associated with reduced PPU, interacting with content creators did not moderate the effect of emotion regulation on the relationship between loneliness and PPU. Our findings support the hypothesised model, suggesting that emotion regulation difficulties mediate the relationship between loneliness and PPU. The identified mediating role of emotion regulation difficulties provides a foundation for further investigations into the underlying mechanisms connecting emotional well-being and problematic pornography use, offering potential directions for future research.

## Highlights

• Loneliness was positively associated with Problem Pornography Use (PPU)
• Emotion regulation difficulties mediated the relationship between loneliness and PPU.
• Interaction with content creators was negatively associated with PPU, but did not moderate effects.

## 1 Introduction

Pornography is a widespread global and normative phenomenon (Bőthe et al., 2021, Grubbs et al., 2019, Grubbs et al., 2019), engendering much debate around its use. Individuals use pornography to serve a range of different functions − most commonly arousal (D’Orlando, 2011), and to enhance masturbation (Solano et al., 2020), but also as a means of education, exploring curiosities (Rissel et al., 2017), seeking relationships (Grubbs et al., 2019, Grubbs et al., 2019), and to serve as a means of stress release or distraction (Bőthe et al., 2021). However, pornography use has also been linked to a range of negative consequences, including low self-esteem (Schneider, 2000), reduced productivity (Young, 2004), low mood and anxiety (Philaretou et al., 2005), and reductions in sexual satisfaction within relationships (Poulsen et al., 2013). Recent attention has therefore turned its attention to *problematic pornography use* (PPU) − the tendency to use pornography excessively or compulsively, reflecting an inability to control its use despite the user’s attempts (Bőthe et al., 2018, Grubbs et al., 2018). Accordingly, research has accumulated in relation to identifying the key diagnostic features of PPU, to inform potential interventions (Chen and Jiang, 2020, Fernandez and Griffiths, 2021). Problematic pornography use shares many similarities with other addictive behaviours (Griffiths, 2012), observing a range of neurobiological (Gola et al., 2016, Love et al., 2015), cognitive (Brand et al., 2016), and behavioural mechanisms (Elmquist et al., 2016, Laier et al., 2013).

Recent research by Bőthe et al. (2020) note that frequency of use appears unrelated to PPU, and that PPU may be better explained by examining individual differences and social and societal contexts. One such context may be the experience of *loneliness*, defined as the aversive emotional state that arises when there is a disconnect between one's desired and perceived interpersonal relationships, often due to a lack of both quality and quantity in meaningful connections (Peplau & Perlman, 1982). Loneliness is quickly emerging as an immediate public health concern, with a recent *meta*-analysis by Rico-Uribe et al. (2018) finding it increases the risk of all-cause mortality by 22 %. Accordingly, the World Health Organization (WHO) have established a Commission on Social Connection to address the health and wellbeing impacts associated with loneliness (WHO, 2023). The relationship between loneliness and PPU may be bidirectional, with studies showing loneliness may drive individuals to engage in PPU as a coping strategy, and PPU may exacerbate loneliness where it contributes to relationship difficulty (Mestre-Bach & Potenza, 2023).

Another correlate of PPU that has relevance to loneliness is difficulty in emotion regulation – a set of processes underlying the experience, expression, understanding, and management, of different types of emotions (Gross & Muñoz, 1995). Recent research by Cardoso and colleagues (2022, 2023) found that both loneliness and difficulties in emotion regulation predicted PPU, and that loneliness mediates the effect of difficulties in emotion regulation on PPU. While the directionality of the relationship between loneliness and emotion regulation has not been well-explored, research has indicated bidirectional associations between loneliness and affective disorders (Santini et al., 2020), indicating that loneliness may shape affective experiences, and that emotions may precipitate loneliness. Similarly, Preece et al. (2021) suggest that individuals experiencing high levels of loneliness may wish to form social connections to serve interpersonal needs; however, paradoxically, may respond to the experience of negative emotion maladaptively (e.g., avoiding social contact), which would perpetuate loneliness (Preece et al., 2021). One theoretical perspective is that loneliness engenders a feeling of “unsafeness”, impairing emotional and cognitive processes (Cacioppo & Hawkley, 2009). Indeed, Kearns and Creaven (2016) have suggested that emotion regulation may be a mechanism “by which individuals experience loneliness” (p. 70). It is therefore plausible to position emotion regulation difficulties as a potential explanatory variable for the relationship between loneliness and PPU – that is, loneliness creates an aversive emotional stimulus, and when an individual experiences difficulties in managing that, they may begin to engage in problematic use.

It would be remiss to investigate the relationship between loneliness and pornography use without considering the nature of pornography consumption in the modern era, given pornography use has become more acceptable over time (Price et al., 2016), to the extent that online pornography use is now considered an element of online sexual activity more broadly (Ballester-Arnal et al., 2021). Notably, the explosion of online pornographic content platforms that include paid and free subscriptions (e.g., *OnlyFans*, *JustForFans*, *FanCentro*, *fansly* and *ismygirl*.) reflects consumer demand for a form of intimate, authentic, and accessible connection, facilitated by instant messaging or live-streaming that allows for a range of ways in which to interact with the content creator (Easterbrook-Smith, 2022, Van der Nagel, 2021). Modern pornography use is becoming increasingly complex, affording opportunities for individuals to engage in pornography use alone or with others (e.g., a content creator), and consume pornography synchronously (i.e., live) or asynchronously. Therefore, it is important to contextualise the modern pornography landscape when considering PPU, especially if individuals are drawn to PPU through their difficulties managing emotional experiences that stem from loneliness. While there is a dearth of research on pornography users who access content creation platforms, recent research has found that users and nonusers exhibit comparable sexual attitudes (Litam et al., 2022). However, based on interviews with OnlyFans creators, Cardoso et al. (2023) suggest it is not merely pornographic content that is sought, but also a sense of connection and intimacy. It is plausible that an individual who experiences loneliness may seek out an online interaction with a content creator to ameliorate feelings of loneliness through that interaction.

### 1.1 The present study

**Fig. 1:**
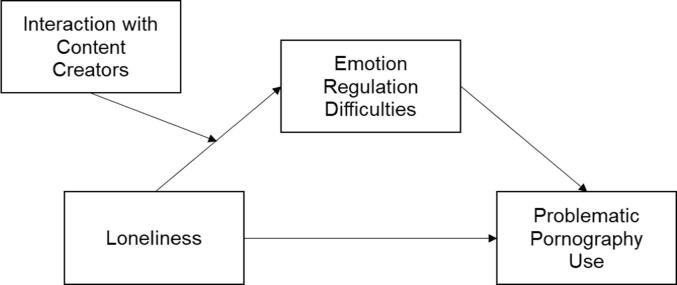
Moderated mediation model.

This study sought to explore the interrelationships between loneliness, emotion regulation difficulties, and PPU. It was hypothesised that (a) loneliness would be positively associated with PPU; (b) loneliness would also be positively associated with emotion regulation difficulties, and; (c) emotion regulation difficulties would be associated with PPU. Accordingly, it was hypothesised that emotion regulation difficulties would mediate the relationship between loneliness and PPU, and, that interaction with a pornographic content creator would moderate the effect of loneliness on emotion regulation difficulties. Fig. 1 depicts the associated moderated mediation model.

## 2 Method

### 2.1 Participants

The sample comprised 213 respondents aged between 18 and 79 years (*M* age = 35.57 years, *SD* = 16.90; 77.50 % men) who completed all standardised measures in the survey and passed at least two of three random attention checks used throughout the survey (e.g., “please select *Almost Always*”). Regarding respondents' relationship status, about half of the participants reported to be in a relationship (*n* = 108, 48.60 %). Table 1 provides information on pornography use behaviours for the sample. Most of the participants (*n* = 65, 30.50 %) reported spending from 16 to 25 min using pornography on each occasion, and 83.60 % indicated they use pornography by themselves. Thirty-seven (17.40 %) of the participants indicated that they interact with a pornographic content creator.

**Table 1:** Sample pornography use behaviours (N = 213).

Behaviour	*n*	%
Frequency of pornography use		
Never or rarely	28	13.1
Monthly	52	24.4
Weekly	78	36.6
Daily	55	25.8
Duration of pornography use		
1-5 min	16	7.5
6-15 min	56	26.3
16-25 min	65	30.5
26-35 min	34	16
36-45 min	20	9.4
>45 min	21	9.9
Platform		
Videos on internet pornography website/ platform/ app	193	90.6
Photos (or images) on internet pornography website/ platform/ app	181	85
Pornography films or movies (e.g., on DVD, TV, Cable, VCR)	104	48.8
Written pornographic literature (Offline or Online)	116	54.5
Pornography games or virtual reality	80	37.6
Pornography Magazines, Pornography Comic Books	100	46.9
Live show / Performance (In person)	65	30.5
Live show / Performance (online)	73	34.3
Other	51	23.9
Pornography interaction		
Do you interact with the pornography star / content creator? YES	37	17.4
Do you interact with the pornography star / content creator? NO	176	82.6

### 2.2 Procedure

The study was conducted in accordance with the Declaration of Helsinki and was approved by the authors' University Human Research Ethics Committee (Approval No. H22109). The participants for this study were recruited via social media advertising on Facebook, Instagram, and TikTok, as well as through the use of posts on LinkedIn, Twitter, and Reddit. Respondents were eligible to enter the survey if they were English-speaking Australians (Permanent Residents or Citizens), aged 18 years or older, who use or have used pornography. All participants were presented with a plain language statement that advised them of the nature and aim of the study, their rights to withdraw at any stage, and provided electronic informed consent prior to proceeding with the questionnaire. Only individuals who were at least 18 years old and permanently residing in Australia were invited to participate. Data were collected between December 2022 and March 2023.

### 2.3 Measures

#### 2.3.1 Problematic pornography use

The Problematic Pornography Consumption Scale (PPCS; Bőthe et al., 2018) is an 18-item self-reported measure developed to assess problematic pornography use. Participants rated items concerning their consumption of pornography (e.g., *I released my tension by watching porn*), using a Likert response scale from 1 (*never)* to 7 (*all the time*). A cut-off of 76 points identifies a possible problematic pornography use, exceeded by 15 % of the sample. The internal consistency of the PPCS original study was excellent (*α* = .93) as in the present study (*α* = .95).

#### 2.3.2 Loneliness

The University of California Los Angeles Loneliness Scale − Revised (UCLA-R; Russell et al., 1980) assesses feelings of loneliness, social isolation, and lack of connectedness. The 20 items reflect both emotional and social dimensions of loneliness (e.g., *My interests and ideas are not shared by those around me*). Participants respond to these on a Likert scale ranging from 1 = (*never*) to 4 (*always*). A higher total score indicates greater loneliness. The internal consistency of the measure in the original study (Cronbach's = .94) and current study (0.90) indicates excellent internal consistency.

#### 2.3.3 Difficulties in emotion regulation

The Difficulties in Emotion Regulation Scale (DERS; Gratz & Roemer, 2004) is a 36-item self-report measure, developed to assess emotion regulation difficulties. Participants are asked to indicate how often each statement (e.g., *When I’m upset, I believe that I will remain that way for a long time*) using a five-point Likert scale ranging from 1 (*almost never*) to 5 (*almost always*). The items can be summed to generate a total scale score (e.g., Osborne et al., 2017), with higher scores indicating greater emotion regulation difficulties. The internal consistency of was acceptable for the present study (*α* = .95).

#### 2.3.4 Pornography use behaviors

Participants completed a series of questions related to pornography use behaviours (i.e., frequency of pornography use, duration of pornography use per session, preferred pornography modality, and whether or not they interact with a pornographic content creator).

### 2.4 Data preparation and analytical plan

Data were analysed using SPSS 28.0 and conducted in three steps. The first step included the descriptive analyses and the examination of the variable characteristics of loneliness, emotion regulation difficulties, interaction with the pornography content creator and problematic pornography use. The second step consisted of correlation analyses to examine associations between variables. The third step consisted of testing the proposed model using the SPSS PROCESS macro (Hayes, 2017), which tests mediating, moderating and moderated mediation effects in a single model. Model 7 was used to test the hypotheses underlying the model. The simple mediating effect analysis (emotion regulation difficulties) is used to examine how the predictor variable (loneliness) influences the outcome variable (problematic pornography use), whereas the simple moderating effect analysis (interaction with the pornography content creator) becomes operative when the relationship between the predictor and outcome variable changes depending on the level of the moderator (Hayes, 2017). This analysis therefore allowed for examination of the mediating effect of emotion regulation difficulties in relation to the association between loneliness and problematic pornography use, and under which conditions this mediating effect is significant.

## 3 Results

The variables were inspected for univariate skewness and multivariate outliers. All the continuous variables were not substantially skewed (loneliness = .06, emotion regulation difficulties = .29, and problematic pornography use = .79). Cook’s distance was used to check for the influence of multivariate outliers, with no influential cases detected as a concern. Similarly, screening revealed no concerns with multicollinearity, with the tolerance values well above 0.20 for each variable (minimum tolerance = .69).

Table 2 displays inter-correlations, means and standard deviations of the variables of interest. As shown, there was a positive correlation between the predictor variable (loneliness) and the outcome variable (problematic pornography use). Similarly, the potential mediator (emotion regulation difficulties) was correlated with problematic pornography use, thus satisfying the requirements for further testing of the mediation effects (Hayes, 2017). Age was negatively correlated with problematic pornography use and difficulties with emotion regulation. Gender (i.e., male) was positively correlated with problematic pornography use, loneliness, and emotion regulation difficulties. Therefore, age and gender were included as covariates in the moderated mediation model. Lastly, interaction with the pornographic content creator was negatively associated with a higher degree of problematic pornography use (*p* < 0.001).

**Table 2:** Correlations and Descriptive Statistics for Study Variables.

Scale	1	2	3	4	5	*M*	*SD*
1. PPU	.95					50.28	22.54
2. ERD	.53^***^	.95				87.58	25.73
3. Loneliness	.37^***^	.56^***^	.92			45.62	12.02
4. Age	−.20^**^	−.34^***^	.06	−		35.5	16.90
5. Gender	−.31^***^	−.14*	−.28^***^	−.12	−		
6. ICC	−.21^***^	−.13	−.09	.10	−.02		

*Note*. PPU = problematic pornography use; ERD = emotional regulation difficulties; ICC = interaction with content creator. Cronbach's α are included along the principal diagonal; Omega coefficients were equal to these values. *p < 0.05, **p < 0.01; ***p < 0.001.

### 3.1 Moderated mediation model

**Fig. 2:**
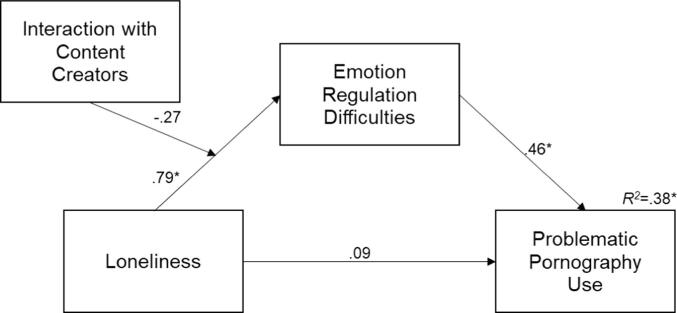
Moderated Mediation Model controlling for age and gender; coefficients are standardised (^∗^*p* < 0.001).

The SPSS PROCESS macro (version 4.2) by Hayes (2017) was used to assess the moderated mediation model (model 7), generating 5000 bootstrapped 95 % Confidence Intervals. The model revealed a significant indirect effect between loneliness and problematic pornography use via emotion regulation, rendering the direct path between loneliness and problematic pornography use non-significant (*β* = .09, 95 % *CI* [-0.11,.43]), and indicating emotion regulation completely mediated the relationship between loneliness and problematic pornography use. However, the mediation effect was not moderated by interaction with the pornography content creator (*β* = -0.57, 95 % *CI* [-1.31,.18]). Table 3 includes the unstandardised parameter estimates for the moderated mediation model; Fig. 2 includes the standardised parameter estimates for the model.

**Table 3:** Results of the Moderated Mediation Analyses.

Predictors	On DERS				On PPCS			
	*β*	*SE*	*p*	95 % *CI*	*β*	*SE*	*p*	95 % *CI*
Age	−0.55	0.08	<.001	[-0.71, −0.40]	−0.15	0.09	.089	[-0.31, 0.02]
Gender	−2.85	3.58	.427	[-9.91, 4.21]	−13.78	3.40	<.001	[-20.49, −7.07]
Loneliness	2.27	0.73	.002	[0.83, 3.70]	0.16	0.14	.232	[-0.11, 0.43]
ICC	23.41	18.50	.207	[-13.07, 59.91]				
ERD					0.41	0.07	<.001	[0.28, 0.53]
Loneliness x ICC	−0.57	0.38	.134	[-1.32, 0.18]				
*R^2^*	0.45				0.38		<.001	
*F*	32.59				30.92			

Note: *N* = 203. Analysis conducted using PROCESS Model 7. ICC = interaction with content creator; ERD = emotion regulation difficulties, PPU = problematic pornography use.

## 4 Discussion

The present study sought to test a moderated mediation model, whereby difficulties in emotion regulation mediated the effect of loneliness on problematic pornography use, and that this would be dependent on whether or not individuals interacted with a pornographic content creator. Correlational analyses indicate positive associations between loneliness, emotion regulation, and PPU, consistent with existing research findings (e.g., Cardoso et al., 2022, Mestre-Bach and Potenza, 2023). The hypothesised mediation of the loneliness to problematic pornography use path was supported, indicating that emotion regulation completely accounted for the association with these variables. Contrary to hypotheses, the moderating effect of interacting with a pornography content creator, was not statistically significant, and the moderated mediation model was not supported. However, complete mediation of the effect of loneliness on PPU by emotion regulation was observed. Taken together, the findings help clarify the role of emotion regulation difficulties in relation to loneliness and problematic pornography use.

The mediation effect observed indicates that individuals experiencing loneliness may turn to problematic pornography use as a consequence of difficulty in regulating emotion. This is consistent with the findings of Laier and Brand (2017), who used the pornography consumption inventory (PCI; Reid et al., 2011) to ascertain the effects of mood and arousal on internet pornography use. The authors found that pornography use correlated with daily stress, and emotional avoidance as measured in the PCI (i.e., “I use it to avoid feeling uncomfortable or unpleasant emotions”). Specifically, individuals use pornography as a means to help regulate mood that may be influenced by the experience of stress. While emotional avoidance is considered a type of motive in the PCI, conceptually, difficulties in emotional regulation could lead individuals to pursue motives consistent with emotional avoidance. The regulation of aversive states can impact the choice to engage in pornography consumption, and its compensatory effects may alleviate negative emotions, strengthening the thought patterns associated with the rewarding aspects of pornography use and escalating this behavior as a coping mechanism (Brand et al., 2016). In their assessment of the bidirectional relationship between loneliness and excessive pornography use, Butler et al. (2019) position excessive pornography use within a behavioural addiction framework, where individuals become drawn to pornography as a maladaptive form of coping strategy that may worsen loneliness in the longer term.

Despite the absence of moderated mediation effect, the correlation observed between interacting with a content creator and PPU warrants some speculation. Individuals may engage in interaction in a way that alleviates the emotional experience of loneliness, though further investigation is required to identify if this could be considered adaptive. Recent research has examined the notion of “digital sex work” (i.e., sex work within the platform economy) and digital intimacy. Swords et al. (2023) use the term *interpenetration* to describe the phenomenon whereby pornographic content creators (i.e., sex workers) engage with consumers across multiple platforms (e.g., social media platforms), in part to increase sociality. As such, interacting with a content creator may serve as a buffer against problematic pornography use. While speculative, it could be that interaction serves as a form of interpersonal emotion regulation, that is thought to be more prominent when an individual’s intrapersonal (i.e., internal) emotion regulation is deficient (Hofmann et al., 2016). Whether or not this interaction can be interpreted as a form of interpersonal emotion regulation would need to be investigated in future problematic pornography use research. Given the increasing popularity of content creation platforms, as noted by Litam et al. (2022), a more comprehensive exploration of this dynamic, including the use of qualitative research methods, could also yield valuable insights for future research and interventions in this area.

In terms of the practical significance of these findings, the mediation model indicates that attempts to reduce problematic pornography use could consider loneliness and emotion regulation as potential targets for intervention. A recent *meta*-analysis investigating the effective characteristics of loneliness interventions revealed those that promote social connection, friendship, or community integration, as well as psychological strategies such as mindfulness, psychoeducation, coping, and, cognitive behaviour therapy, appear effective (Morrish et al., 2023). Psychological interventions outperform controls, with *meta*-regression analyses revealing that effects are not moderated by age nor sex – however, theoretical approaches are broad and lack clear theoretical alignment with loneliness (Hickin et al., 2021). This combination of findings provides preliminary support for the conceptual premise that clinicians may be able to assist clients struggling with PPU by addressing their emotion dysregulation or lack of strategies for emotion regulation (Barlow et al., 2020). This assistance may help break the detrimental cycle where increased pornography use leads to decreased social interactions (Bőthe et al., 2018, Bőthe et al., 2020). Given the high reliance on the internet as a source of pornographic content, emotion regulation interventions that capitalise on digital technology are also worth consideration in the problematic pornography use context. For example, just in time adaptive interventions (JITAIs), ecological momentary interventions (EMIs), and other internet-based adaptations of existing emotion regulation interventions may prove effective (Bettis et al., 2022, Colombo et al., 2019). Such digital or internet-based interventions may also be especially relevant for individuals experiencing problematic pornographic use who may not present to traditional therapeutic contexts until they experience a range of consequences stemming from excessive use (Kraus et al., 2016), and those experiencing a high degree of loneliness and social withdrawal – potentially related to excessive use (Palazzolo et al., 2020). Such an approach may equip individuals with improved emotion regulation skills, reducing their inclination to engage in PPU even when experiencing loneliness (Levin et al., 2019, Preece et al., 2021).

### 4.1 Limitations and future research directions

The findings of this study need to be considered in light of a range of limitations. While it adopted Cacioppo and Hawkley’s (2009) theorised approach for the experience of loneliness as resulting in distressing emotions, research on bidirectional associations indicates that longitudinal research is a priority for improving understanding of the causal relations between loneliness, emotion regulation, and problematic pornography use. Similarly, while the focus on pornographic content creators was somewhat peripheral to the main hypothesis, the measurement of interactivity warrants further refinement. It may be that a binary measure of this was not sensitive enough to detect a moderation effect. Future studies might consider adopting a method of measurement that provides continuous scales, that allow the collection of more nuanced data in relation to (1) the frequency with which an individual interacts with a content creator when using pornography (e.g., not at all, to exclusively); (2) the nature of that interaction – for instance, whether it is synchronous (i.e., during a live performance) or asynchronous (e.g., contacting creators to obtain recorded material). This would lead to a better characterisation of the nature of interaction that could be important in relation to establishing its effects on problematic pornography use. While religious affiliation or morality were not the focus of the present study, such factors are often incorporated in pornography use research (e.g., Floyd & Grubbs, 2022). Future research may consider including religious affiliation or morality as individual differences variables to investigate their relationship with factors determining problematic use, such as interaction with a content creator, or pornography use within a relationship context. Further although 15 % of the participants exceeded the cut-off score proposed for differentiating problematic from non-problematic pornography use, future research should attempt to replicate these findings in treatment-seeking or high-risk samples to better understand the perpetuating role of loneliness and difficulties with emotion regulation in PPU.

In summary, this study attempts to clarify the role of emotion regulation in relation to the associations between loneliness and problematic pornography use. This highlights the importance of addressing loneliness and emotional regulation as critical components that will help to understand and intervene in problematic pornography use. Given the modern pornography landscape and preponderance of pornography on digital media platforms, there is merit in further investigating the nature and role interactions with pornographic content creators play in relation to helping individuals regulate or cope with their emotions, and how this relates to problematic use.

## CRediT authorship contribution statement

**Maria Vescan:** Writing – original draft, Project administration, Methodology, Investigation, Formal analysis, Data curation, Conceptualization. **Mal Flack:** Writing – review & editing, Validation, Supervision, Methodology, Conceptualization. **Kim M Caudwell:** Writing – review & editing, Supervision, Investigation, Conceptualization.

## Declaration of competing interest

The authors declare that they have no known competing financial interests or personal relationships that could have appeared to influence the work reported in this paper.

## Data Availability

Data will be made available on request.
